# Emergence of Transferable Plasmid Coharboring *mcr-1.1* and *bla*_CTX-M−55_ Genes in Multidrug‐Resistant *Escherichia coli* From Mink

**DOI:** 10.1155/tbed/4811804

**Published:** 2026-06-17

**Authors:** Zizhu Lin, Weiqi Guo, Jiangang Hu, Zhiyang Wang, Beibei Zhang, Xinyu Wang, Haoheng Peng, Jingjing Qi, Mingxing Tian, Yanqing Bao, Lei Deng, Xue Bai, Haihua Li, Shaohui Wang

**Affiliations:** ^1^ Shanghai Veterinary Research Institute, Chinese Academy of Agricultural Sciences, Shanghai, 200241, China, caas.cn; ^2^ Tianjin Key Laboratory of Agricultural Animal Breeding and Healthy Husbandry, College of Animal Science and Veterinary Medicine, Tianjin Agricultural University, Tianjin, 300384, China, tjac.edu.cn; ^3^ Key Laboratory of Special Animal Epidemic Disease, Ministry of Agriculture and Rural Affairs, Institute of Special Animal and Plant Sciences, Chinese Academy of Agricultural Sciences, Changchun, 130112, China, caas.cn

**Keywords:** *bla*_CTX-M−55_, *Escherichia coli*, *mcr-1.1*, multidrug resistance, whole genome sequencing

## Abstract

Multidrug‐resistant (MDR) bacteria have reduced the effectiveness of antimicrobial agents and limited available treatment options in both human and animal settings. Antimicrobial resistance (AMR) in animal‐derived *Escherichia coli* (*E. coli*) has become increasingly common, with many isolates showing resistance to multiple classes of antimicrobials. However, compared with other animal sources, resistance characteristics of *E. coli* from mink remain less well understood, particularly in terms of genomic features. In this study, we identified a MDR *E. coli* strain, EC0D1, isolated from the lung tissue of a farmed mink that succumbed to hemorrhagic pneumonia (HP) in China. Antimicrobial susceptibility testing indicated that EC0D1 exhibited resistance to β‐lactams, fluoroquinolones, aminoglycosides, tetracyclines, and polymyxins but was sensitive to carbapenems and tigecycline. Whole genome sequencing showed that EC0D1 contains a single chromosome and eight plasmids. Among these, plasmid pEC0D1‐3 coharbored *bla*_CTX-M−55_ and *mcr-1.1*, while pEC0D1‐2 carried *tet*(A) and *floR*, together explaining the observed MDR phenotype. Phylogenetic analysis classified EC0D1 as sequence type ST457 and demonstrated a close genetic relationship with several human clinical isolates. Comparative genomic analysis further revealed that plasmids pEC0D1‐2 and pEC0D1‐3 shared high sequence similarity with plasmids previously identified in avian‐derived *E. coli* and human‐derived *Klebsiella pneumoniae* isolates. Conjugation assays confirmed that plasmids carrying *bla*_CTX-M−55_, *mcr-1.1*, *tet*(A), and *floR* were transferable to different bacterial recipients. The emergence of transferable resistance determinants in mink‐associated *E. coli* suggests a potential role in the transmission of resistance genes between human and animal hosts.

## 1. Introduction

The widespread use of antimicrobial agents has substantially reduced morbidity and mortality in both humans and animals; however, their prolonged and sometimes inappropriate use has accelerated the emergence of multidrug‐resistant (MDR) Enterobacteriaceae [1]. Among Enterobacteriaceae, *Escherichia coli* (*E. coli*) is a ubiquitous intestinal commensal of humans and animals; however, pathogenic lineages carrying distinct virulence determinants are capable of causing diverse diseases in livestock and poultry [2]. In cattle, pathogenic *E. coli* is a major cause of calf diarrhea and mastitis [3]; in pigs, cases of postweaning diarrhea and edema disease are commonly observed [4]; and in poultry, it causes colibacillosis, typically manifested as airsacculitis, pericarditis, and septicemia [5]. Although less frequently reported, *E. coli* has also been implicated in severe respiratory infections in mink, including hemorrhagic pneumonia (HP), a disease characterized by acute onset and high mortality [6, 7]. These disease manifestations emphasize the role of *E. coli* as a pathogen of concern in both veterinary and public health settings, particularly in the context of antimicrobial resistance (AMR).

*E. coli* is widely recognized as a key reservoir and vector for AMR genes (ARGs), and it is routinely employed as a sentinel organism in resistance monitoring programs [8, 9]. Strains producing extended‐spectrum β‐lactamases (ESBLs) display reduced susceptibility to critically important antimicrobials, which constrains available treatment options in both clinical and veterinary settings and promotes the spread of resistant bacteria along the food chain [10]. Among ESBL determinants, *bla*_CTX-M−55_—a derivative of *bla*_CTX-M−15_ characterized by a Val77Ala substitution—has been reported to confer increased hydrolytic efficiency toward cephalosporins [11]. In China, *bla*_CTX-M−55_ ranks among the dominant ESBL genotypes identified in isolates from humans and animals, suggesting frequent interhost transmission [12]. Increasing evidence indicates that such resistance genes can circulate across animal, environmental, and human reservoirs via the food chain, contaminated farm environments, and the agricultural application of manure, which may increase the risk to public health [13, 14].

Colistin is considered a critical last‐line agent for managing infections caused by carbapenem‐resistant Enterobacteriaceae; however, its clinical utility has been increasingly compromised by the spread of plasmid‐borne *mcr* genes, since the first identification of *mcr-‍1* in China in 2015 [15], a series of related determinants (*mcr-1* to *mcr-10*) have subsequently been identified, among which *mcr-1* remains the most widely distributed variant [16]. Notably, the concurrent presence of *mcr-1* and ‍‍*bla*_CTX-‍M−55_ in *E. coli* has been repeatedly documented in isolates derived from poultry, swine, and human sources, suggesting that these genes can spread across different hosts and environments [17]. The presence of these resistance determinants in the same strain further limits treatment options during severe infections; in veterinary settings, where alternative antimicrobials are limited, treatment choices may be further constrained. In addition to these clinically significant resistance determinants, *tet*(A) and *floR*, conferring resistance to tetracyclines and phenicols, are also of concern due to their frequent colocalization with ESBL and colistin resistance genes on transferable plasmids. Mobile genetic elements (MGEs), including plasmids, insertion sequences (ISs), and transposons, are central to the acquisition and dissemination of these genes [18]. Because these resistance genes are often embedded in transferable mobile elements, identifying their plasmid context is important for understanding how they spread.

In this study, a MDR *E. coli* strain (EC0D1) from mink was characterized, and its resistance determinants were analyzed for their genetic basis and transferability. Whole genome sequencing identified multiple ARGs, including *mcr-1.1*, *bla*_CTX-M-‍55_, *tet*(A), and *floR*. Conjugation assays further demonstrated that these resistance determinants were located on transferable plasmids, conferring resistance to colistin, extended‐spectrum cephalosporins, tetracyclines, and phenicols. Together, these findings elucidate the genetic context and mobility of clinically important resistance genes and indicate their potential involvement in the cross‐species dissemination of AMR.

## 2. Materials and Methods

### 2.1. Bacterial Strains

The extraintestinal pathogenic *E. coli* (ExPEC) strain EC0D1 was isolated from the lung tissue of a mink with HP from a mink farm in northern China. Antimicrobial susceptibility profiling demonstrated that EC0D1 exhibited a MDR phenotype and carried multiple virulence‐associated genes [19], which was selected for further analysis.

### 2.2. Antimicrobial Susceptibility Testing

The antimicrobial susceptibility profiles of the *E. coli* isolates were assessed using the Kirby–Bauer disk diffusion assay, following the recommendations of the Clinical and Laboratory Standards Institute (CLSI). A panel of agents spanning eight antimicrobial categories was included: β‐lactams (ceftriaxone, cephalothin, cefotaxime, and cefuroxime), quinolones (enrofloxacin), sulfonamides (sulfamethoxazole), phenicols (florfenicol), tetracyclines (tetracycline and tigecycline), polymyxins (polymyxin B), nitrofurans (nitrofurantoin), and carbapenems (imipenem, meropenem, and ertapenem).

Minimum inhibitory concentrations (MICs) were measured using the broth microdilution technique in accordance with the recommendations of the CLSI. For the EC0D1 isolate, MIC values for polymyxin B, ceftazidime, cefuroxime, enrofloxacin, chloramphenicol, imipenem, tigecycline, and tetracycline were further evaluated based on the combined interpretive criteria of CLSI and the European Committee on Antimicrobial Susceptibility Testing (EUCAST). All antimicrobial compounds were purchased from Wenzhou Cont Biotechnology Co., Ltd. The reference strain *E. coli* ATCC 25922 served as the quality control.

### 2.3. Detection of ARGs

EC0D1 was examined for the presence of ARGs, including *mcr-1.1* and *bla*_CTX-M−55_, using polymerase chain reaction (PCR) with gene‐specific primers [20]. Each reaction was conducted with appropriate positive and negative controls to ensure reliability. The resulting amplicons were resolved by electrophoresis on 1.0% (w/v) agarose gels, followed by ethidium bromide staining and visualization under ultraviolet light.

### 2.4. Whole Genome Sequencing and Analysis

Genomic DNA was isolated using a commercial purification kit according to the manufacturer’s protocol. The quality, concentration, and integrity of the extracted DNA were evaluated using a NanoDrop spectrophotometer, a Qubit fluorometer, and electrophoresis on 0.35% agarose gels.

Whole genome sequencing was conducted by combining short‐read data generated on the Illumina NovaSeq platform with long‐read data obtained from the Oxford Nanopore system. Hybrid assembly of the sequencing reads was performed using CANU v1.7.1 and HGAP v4 [21]. Genome assembly services were provided by Shanghai Personal Biotechnology Co., Ltd. The resulting contigs were integrated to produce a complete genome, followed by error correction with Pilon to enhance base‐level accuracy [22].

Plasmid sequences were first annotated using the RAST server and subsequently refined through manual curation. Resistance genes, virulence factors, and plasmid replicons were identified using ABRicate with ResFinder, VFDB, and PlasmidFinder databases. Comparative analysis of plasmids among *E. coli* isolates was carried out using the BLAST Ring Image Generator (BRIG v0.95) [23]. Genomic contexts and structural features were visualized with Easyfig v2.2.5 [24].

Genetic relatedness among isolates from different sources was assessed using a core genome multilocus sequence typing (cgMLST)–based dendrogram [25]. In addition, a single‐nucleotide polymorphism (SNP)–based phylogenetic tree was generated to provide higher‐resolution comparisons of genomic variation among the isolates [26].

### 2.5. Conjugation Assay

Conjugation assays were conducted to evaluate the transfer potential of plasmids pEC0D1‐2 and pEC0D1‐3 from the donor strain *E. coli* EC0D1. Conjugation was carried out in Luria‐Bertani (LB) broth at 37°C for 16–20 h, with *E. coli* J53 (sodium azide‐resistant) and *Klebsiella pneumoniae* (*K. pneumoniae*) 293Z (potassium tellurite‐resistant) serving as recipient strains.

Transconjugants were selected on LB agar supplemented with cefotaxime (2 μg/mL), colistin (2 μg/mL), tetracycline (10 μg/mL), or chloramphenicol (4 μg/mL), in combination with sodium azide (150 μg/mL) or rifampicin (50 μg/mL), as appropriate. Transfer frequencies were calculated as the ratio of transconjugants to recipient cells. The presence of resistance genes in transconjugants was confirmed by PCR. Plasmid pEC0D1‐2 was identified by the detection of *tet*(A) and *floR*, whereas pEC0D1‐3 was identified by the detection of *bla*_CTX-M−55_ and *mcr-1.1*.

### 2.6. Nucleotide Sequence Accession Numbers

The complete nucleotide sequences of the chromosome and eight plasmids in the EC0D1 isolate have been submitted to GenBank with the following accession numbers: chr‐EC0D1 (CM129827.1), pEC0D1‐1 (NZ_JBRNXK010000002.1), pEC0D1‐2 (NZ_JBRNXK010000003.1), pEC0D1‐3 (NZ_JBRNXK010000004.1), pEC0D1‐4 (NZ_JBRNXK010000005.1), pEC0D1‐5 (NZ_JBRNXK010000006.1), pEC0D1‐6 (NZ_JBRNXK010000007.1), pEC0D1‐7 (NZ_JBRNXK010000008.1), and pEC0D1‐8 (NZ_JBRNXK010000009.1).

## 3. Results

### 3.1. An *E. coli* Strain EC0D1 Exhibits MDR Phenotype

Antimicrobial susceptibility testing revealed that the EC0D1 isolate exhibited a MDR phenotype (Table 1). This isolate showed high‐level resistance to multiple β‐lactam antibiotics, including ceftazidime (>64 μg/mL), cefuroxime (>64 μg/mL), and cefotaxime (>256 μg/mL). In addition, EC0D1 was resistant to polymyxin B (4 mg/L), tetracycline (16 μg/mL), chloramphenicol (>64 μg/mL), streptomycin (>64 μg/mL), and enrofloxacin (>32 μg/mL). This isolate remained susceptible to imipenem (<0.125 μg/mL) and tigecycline (0.032 μg/mL). EC0D1 showed resistance to a wide range of antimicrobials, particularly cephalosporins and fluoroquinolones. PCR detected multiple ARGs in EC0D1, including *mcr-1.1* and *bla*_CTX-M−55_.

**Table 1:** MICs of antibiotic for EC0D1, J53, 293Z, and transconjugates.

Antibiotics	EC0D1	J53‐pEC0D1‐2	J53‐pEC0D1‐3	J53	293Z‐pEC0D1‐2	293Z‐pEC0D1‐3	293Z
Ceftazidime	>64	<0.125	>64	1	2	>64	2
Cefuroxime	>64	<0.125	>64	<0.125	<0.125	>64	<0.125
Cefotaxime	>256	<0.125	>256	<0.125	<0.125	>256	<0.125
Polymyxin B	4	0.25	2	1	1	4	1
Tigecycline	0.032	0.032	0.032	0.032	0.064	0.064	0.064
Tetracycline	16	16	1	1	16	1	1
Chloramphenicol	>64	>64	2	4	>64	2	2
Imipenem	<0.125	<0.125	<0.125	<0.125	<0.125	<0.125	<0.125
Streptomycin	>64	>64	1	1	>64	2	2
Enrofloxacin	>32	<0.125	<0.125	<0.125	<0.125	<0.125	<0.125

### 3.2. Genomic Features and Phylogenetic Analysis of the ST457 Isolate EC0D1

**Figure 1:**
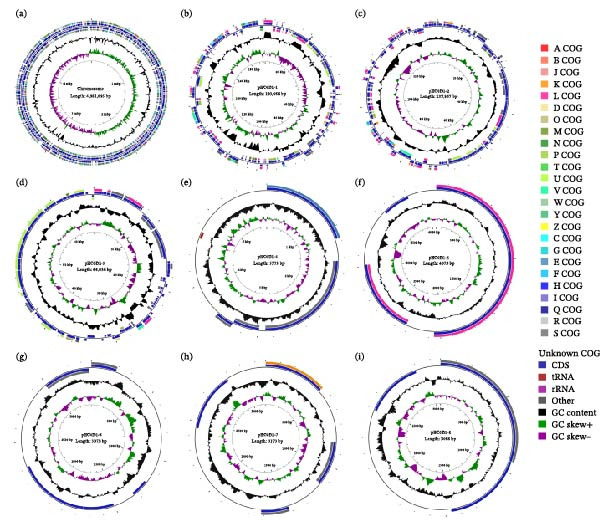
Structure of EC0D1 chromosome and plasmids. (a) Circular chromosome structure of EC0D1. (b–i) Plasmids pEC0D1‐1‐pEC0D1‐8 identified in EC0D1. From outermost to innermost rings, the maps show coding sequences on the forward and reverse strands, antimicrobial resistance genes, virulence‐associated genes, GC content, and GC skew. Different colors represent distinct functional categories based on genome annotation. Resistance genes and virulence‐associated genes are highlighted according to their respective classifications.

The complete genome of *E. coli* strain EC0D1 consisted of a circular chromosome of 4,981,095 bp with a GC content of 50.46% and eight plasmids ranging from 3066 to 193,068 bp in size (Figure 1). The chromosome encoded 4626 predicted open reading frames (ORFs), whereas the plasmids encoded between 3 and 205 ORFs. The GC content of the plasmids ranged from 42.54% to 55.29% (Table 2).

**Table 2:** Genome features and resistome of EC0D1.

Feature	Chromosome	pEC0D1‐1	pEC0D1‐2	pEC0D1‐3	pEC0D1‐4	pEC0D1‐5	pEC0D1‐6	pEC0D1‐7	pEC0D1‐8
Size (bp)	4,981,095	193,068	137,957	66,034	5773	4073	3373	3173	3066
GC content (%)	50.46	49.73	53.77	42.54	45.14	51.39	55.29	46.83	52.28
ORF number	4626	205	180	88	4	3	5	4	3
Inc Group	NA	IncFIC (FII) IncFIB	IncB/O K Z IncX1	IncI2	ColRNAI	NA	NA	ColRNAI	NA
Resistome	—	—	—	—	—	—	—	—	—
Beta‐lactams	*bla*_OXA−1_	—	—	*bla*_CTX-M−55_	—	—	—	—	—
Aminoglycosides	*aac*(*3*)‐*IV* *aph*(*6’*)‐*Ib-cr* *ant*(*3”*)‐*IIa* *aph*(*4*)‐*Ia*	—	*ant*(*3”*)‐*IIa* *aph*(*3”*)‐*Ib* *aph*(*6*)‐*Id*	—	—	—	—	—	—
Phenicol	—	—	*floR*	—	—	—	—	—	—
Tetracycline	—	—	*tet*(*A*)	—	—	—	—	—	—
Sulfonamide	*sul1* *sul3*	—	*sul2*	—	—	—	—	—	—
Polymyxin B	—	—	—	*mcr-1.1*	—	—	—	—	—

Plasmid replicon typing revealed that EC0D1 harbored multiple incompatibility groups, including IncFIC(FII), IncFIB, IncB/O/K/Z, IncX1, IncI2, and ColRNAI. ARG analysis indicated that the chromosome carried *bla*_OXA−1_, *aac*(*3*)‐*IV*, *aph*(*6’*)‐*Ib*, *ant*(*3”*)‐*IIa*, *aph*(*4*)‐*‍Ia*, *sul1*, and *sul3*. Plasmid pEC0D1‐3 simultaneously carried *bla*_CTX-M−55_ and *mcr-1.1*, whereas plasmid pEC0D1‐2 harbored multiple resistance determinants, including *floR*, *tet*(A), *sul2*, and *ant*(*3”*)‐*IIa*. Several plasmids (pEC0D1‐4 and pEC0D1‐8) lacked identifiable resistance genes.

**Figure 2:**
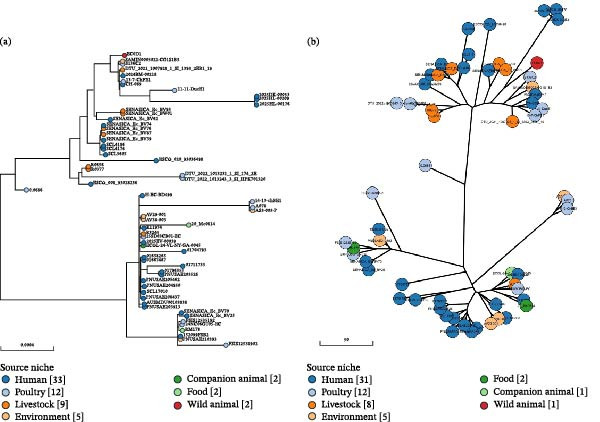
Phylogenetic analysis and host source distribution of *Escherichia coli* isolates from multiple origins. (a) SNP‐based phylogenetic tree of ST457 *E. coli* isolates based on core genome single‐nucleotide polymorphisms. The node colors represent the source of bacterial isolation. (b) cgMLST‐based phylogenetic tree constructed using the EnteroBase *E. coli* core genome multilocus sequence typing scheme. The node colors represent the source of bacterial isolation.

The EnteroBase *E. coli* cgMLST framework was applied to classify genomes into single‐linkage hierarchical clusters (HCs) across 13 predefined resolution levels, spanning from HC0 (genomes with no allelic variation) to HC2350 (genomes differing by up to 2350 alleles). In parallel, SNP‐based analysis was conducted to achieve finer‐scale phylogenetic resolution among the isolates. The cgMLST analysis showed that EC0D1 clustered within a diverse international collection of ST457 isolates and was closely related to several human‐derived *E. coli* strains including the United States (*n* = 19), Mexico (*n* = 9), the United Kingdom (*n* = 6), Bangladesh (*n* = 4), Chile (*n* = 3), and Vietnam (*n* = 2), along with additional strains from other countries. Regarding source distribution, 31 isolates were derived from humans, 12 from poultry, eight from livestock, five from the environment, and two from food samples (Figure 2). These isolates were distributed across a broad geographic range and multiple sources.

Consistent with the cgMLST clustering, SNP‐based phylogenetic analysis further divided the isolates into four major groups. The farmed mink isolate EC0D1, obtained in this study, clustered with several human clinical strains, forming a distinct and well‐supported branch (Figure 2). EC0D1 exhibited a high degree of genetic relatedness to these human isolates, indicating a close evolutionary relationship with the strains of human origin.

**Figure 3:**
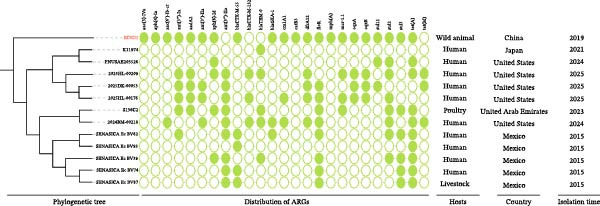
Phylogenetic tree of the strain EC0D1 and related reference genomes retrieved from the NCBI database. Green circles indicate the presence of antimicrobial resistance genes.

To investigate the evolutionary relationship of EC0D1, a phylogenetic analysis was conducted using *E. coli* genomes retrieved from the NCBI nr database (Figure 3). The results showed that EC0D1 clustered with isolates from diverse sources, including human, animal, and environmental origins. The distribution of ARGs further revealed that a considerable proportion of the analyzed isolates carried multiple resistance determinants. EC0D1 exhibited a resistome profile comparable to that of several clinically associated strains, suggesting the presence of shared resistance features. These results show that resistance genes are widely distributed across different hosts and environments.

### 3.3. Plasmid pEC0D1‐2 Carries Multiple Resistance Genes Within a Conserved Multidrug Resistance Region

**Figure 4:**
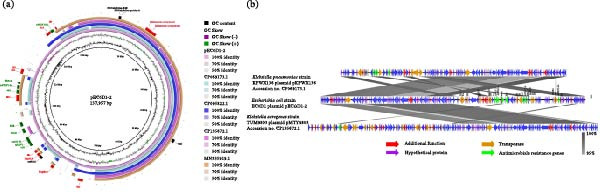
Genomic architecture and comparative genomics of plasmid pEC0D1‐2. (a) Comparison of *floR-tet*(*A*)‐harboring IncB/O/K/Z‐IncX1 plasmid pEC0D1‐2 with CP069173.1, CP095822.1, CP135672.1, and MN335919.1. Red color indicates insert sequences and transposase, green color indicates antimicrobial resistance genes, and black color indicates additional functional proteins. (b) Comparison of the pEC0D1‐2 homologous fragment structure pKPWX136 (Genbank accession number: CP069173.1) and pMTY9803 (Genbank accession number: CP135672.1). Green color indicates antimicrobial resistance genes, orange color indicates insert sequence and transposase, red color indicates additional function protein, and purple color indicates hypothetical protein.

The *tet*(A)‐ and *floR*‐bearing plasmid pEC0D1‐2 (137,957 bp) contained a GC content of ~53.77% and was identified as an IncX1/IncB/O/Z incompatibility group plasmid. Comparative genomic analysis revealed that pEC0D1‐2 shared >95% nucleotide identity and high synteny with plasmids pKPWX136 (GenBank accession number: CP069173.1; *K. pneumoniae*), pJnd12 (GenBank accession number: CP095822.1., *E. coli*), pMTY9803 (GenBank accession number: CP135672.1, *K. pneumoniae*), and pSD‐2 (GenBank accession number: MN335919.1, *E. coli*), originating from Enterobacteriaceae isolates from different hosts and geographic regions (Figure 4). The MDR region of pEC0D1‐2 harbored *tet*(A), *floR*, *sul1*, *aadA2*, *aph*(*3”*)‐*Ib*, and *aph*(*6*)‐*Id*, flanked by multiple ISs (IS26 and IS91) and transposons (Tn3 and Tn5). Compared with the reference plasmids, pEC0D1‐2 exhibited conservation of the MDR backbone but lacked certain accessory genes, such as *dfrA12* in pCP135672.1 and *sul2* in pMN335919.1 (Figure 4). Sequence analysis using OriTfinder identified a complete type IV secretion system (T4SS) together with a coupling protein gene (T4CP), a relaxase gene, and an origin of transfer (oriT) locus within pEC0D1‐2.

### 3.4. Characterization of Plasmid pEC0D1‐3 Coharboring *bla*_CTX-M−55_ and *mcr-1.1* Genes

**Figure 5:**
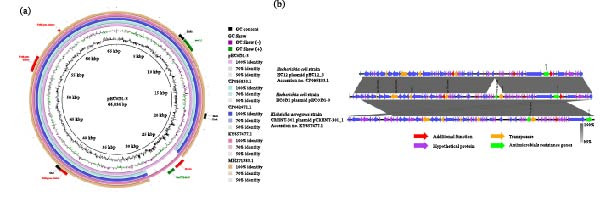
Genomic architecture and comparative genomics of plasmid pEC0D1‐3. (a) Comparison of *mcr-1.1* and *bla*_CTX-M−55_ harboring IncI2 plasmid pEC0D1‐3 with CP095833.1, CP042471.1, KY657477.1, and MH271383.1. Red color indicates insert sequences and transposase, green color indicates antimicrobial resistance genes, and black color indicates additional functional proteins. (b) Comparison of the pEC0D1‐3 homologous fragment structure pEC12_3 (Genbank accession number: CP069173.1) and pCRENT‐301 (Genbank accession number: KY657477.1). Green color indicates antimicrobial resistance genes, orange color indicates insert sequence and transposase, red color indicates additional function protein, and purple color indicates hypothetical protein.

The plasmid pEC0D1‐3 (66,034 bp), carrying *mcr-1.1* and *bla*_CTX-M−55_, has a GC content of 42.52% and belongs to the IncI2 incompatibility group. Comparative genomic analysis revealed that pEC0D1‐3 shared >95% nucleotide identity and high synteny with plasmids pEC12_3 (GenBank accession number: CP095833.1, *E. coli*), pA50‐pC2 (GenBank accession number: CP042471.1, *E. coli*), pCRENT‐301 (GenBank accession number: KY657477.1; a human‐derived *Klebsiella oxytoca*), and pV80 (GenBank accession number: MH271383.1, *E. coli*), identified in Enterobacteriaceae isolates from different hosts and geographic regions (Figure 5).

The MDR region of pEC0D1‐3 contained *mcr-1.1* and *bla*_CTX-M−55_ and was flanked by multiple MGEs, including IS1380, TnAs1, and PAP2 family proteins. Comparative analysis demonstrated that the MDR backbone was conserved among the reference plasmids, although structural variations were observed in the arrangement of the surrounding MGEs (Figure 5). Sequence analysis further identified genes associated with T4SS within pEC0D1‐3.

### 3.5. Multidrug‐Resistance Plasmids pEC0D1‐2 and pEC0D1‐3 Are Transferable Into Multiple Bacterial Recipients

**Figure 6:**
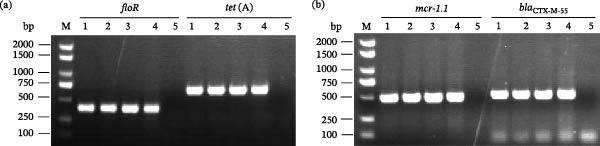
PCR identification of transconjugants carrying resistance plasmids from EC0D1. (a) Lanes 1–2, *K. pneumoniae* transconjugants 293Z‐pEC0D1‐2; lanes 3–4, *E. coli* transconjugants J53‐pEC0D1‐2; lane 5, negative control. (b) Lanes 1–2, *K. pneumoniae* transconjugants 293Z‐pEC0D1‐3; lanes 3–4, *E. coli* transconjugants J53‐pEC0D1‐3; lane 5, negative control.

Conjugation assays w/ere used to assess whether plasmids pEC0D1‐2 and pEC0D1‐3 could be transferred. Transconjugants carrying pEC0D1‐2 were obtained using *E. coli* J53 as the recipient strain and were selected on media supplemented with tetracycline or chloramphenicol in combination with sodium azide. PCR analysis confirmed the presence of *tet*(A) and *floR* in the corresponding transconjugants, and the resistance phenotypes matched the transferred resistance determinants (Figure 6).

Plasmid pEC0D1‐3 was successfully transferred to both *E. coli* J53 and *K. pneumoniae* 293Z recipients. Transconjugants were selected on colistin‐containing media or cefotaxime together with the appropriate selective agents. PCR amplification confirmed the presence of *mcr-1.1* and *bla*_CTX-‍M-‍55_ in the transconjugants. The transfer frequencies of pEC0D1‐3 to *E. coli* J53 and *K. pneumoniae* 293Z were determined to be 10^−2^ and 10^−3^ per recipient cell.

## 4. Discussion

Minks, as both economically significant fur‐bearing animals and wildlife species, are increasingly recognized as potential participants in the global AMR network. Through their frequent interactions with humans, domestic animals, and environmental microbiota, minks increase the likelihood of microbial exchange and may increase the spread of resistant bacteria [27]. Antimicrobials such as tetracyclines, β‐lactams, and sulfonamides are commonly used in mink farming for the prevention and treatment of bacterial infections. Previous studies have shown that antimicrobial consumption in mink production systems is associated with the emergence and persistence of antimicrobial‐resistant bacteria, highlighting the potential selective pressure exerted by antibiotic usage in fur animal breeding environments [28, 29]. *E. coli* is a major zoonotic pathogen worldwide and can lead to urinary tract infections, diarrhea, sepsis, and meningitis in humans and represents an important concern for public health [30]. This study identified a mink‐derived MDR *E. coli* strain EC0D1, coharboring *bla*_CTX-M−55_ and *mcr-1.1* on transferable plasmids.

The AMR phenotype of EC0D1 was generally consistent with its resistance genotype, with the detected resistance genes corresponding well to the observed resistance to β‐lactams, tetracyclines, phenicols, aminoglycosides, sulfonamides, and polymyxins. The resistance profile of EC0D1 is determined by both chromosomal genes and plasmid‐borne elements. Of particular concern is the coexistence of *bla*_CTX-M−55_ and *mcr-1.1*, which compromises susceptibility to extended‐spectrum cephalosporins and colistin, two antimicrobial classes of critical importance for treating severe Gram‐negative infections [31]. This resistance combination is more clinically significant than a broad MDR phenotype alone, as it indicates convergence of last‐line resistance mechanisms within a single strain. EC0D1 carried the highest number of ARGs among the investigated ST457 isolates. However, ARG abundance did not appear to increase with isolation year, suggesting that temporal factors alone may not explain the resistome diversity observed among these strains. A similar co‐occurrence of *mcr-1* and ESBL genes has been increasingly reported in animal‐derived *E. coli* isolates in China, particularly in poultry and swine production systems [15, 17]. In addition, large‐scale epidemiological studies have demonstrated widespread dissemination of *mcr-1* in China across different hosts and environments, supporting the role of cross‐sector transmission in shaping resistance patterns [32].

Compared with previous studies of mink‐derived *E. coli*, which mainly reported resistance to commonly used veterinary antimicrobials such as tetracycline and sulfonamides [28], EC0D1 exhibits a more concerning resistance genotype involving clinically prioritized resistance determinants. This difference may arise from differences in antimicrobial use and local selection pressure or bacterial population structures. Importantly, the detection of *bla*_CTX-M−55_ and *mcr-1.1* in a mink‐associated isolate indicates that resistance genes typically emphasized in livestock and human clinical settings are also present in fur animal populations, thereby expanding the known ecological distribution of these determinants [12].

Phylogenetic analysis demonstrated that EC0D1 belongs to sequence type ST457 and clustered closely with several human clinical isolates in both cgMLST‐ and SNP‐based phylogenetic trees. ST457 has been increasingly recognized as a globally distributed MDR *E. coli* lineage identified in humans, poultry, livestock, and environmental sources [33, 34]. Previous studies have shown that ST457 isolates commonly carry clinically important resistance genes, including *bla*_CTX-M_ and *mcr-1*, indicating their potential role in the dissemination of AMR between animal and human reservoirs [35, 36]. The resistance gene profile observed in EC0D1 was consistent with that reported in other MDR ST457 isolates. In the present study, the close phylogenetic relationship between the mink‐derived isolate EC0D1 and human‐associated strains suggests that ST457 may facilitate the circulation of resistance genes across different hosts and ecological niches, highlighting the potential public health significance of mink‐associated MDR *E. coli* [37].

Conjugation experiments confirmed the strong transferability of resistance plasmids from EC0D1. Both pEC0D1‐2 (carrying *tet*(A) and *floR*) and pEC0D1‐3 (carrying *bla*_CTX-M−55_ and *mcr-1.1*) were successfully transferred to *E. coli* J53 and *K. pneumoniae* 293Z recipients and remained stably maintained. The successful transfer of these plasmids into both *E. coli* and *K. pneumoniae* recipients indicates that EC0D1 carries resistance elements with broad dissemination potential. Transfer across different bacterial species may increase the risk of AMR spreading in clinical and environmental settings, particularly when recipient species such as *K. pneumoniae* are important nosocomial pathogens [38].

Comparative genomic analysis elucidated the structural features and evolutionary relationships of these plasmids. pEC0D1‐2 (IncB/O/K/Z‐IncX1 hybrid) carried *floR* and *tet*(A) within a multidrug resistance region, flanked by IS26, IS91, and Tn3 elements. IncB/O/K/Z plasmids are frequently associated with genes conferring resistance to second‐ and third‐generation cephalosporins and cotrimoxazole [39, 40]. Comparative analysis revealed >97% sequence identity to avian‐origin plasmids such as pCP135672.1 and pMN335919.1, which also harbor *floR-tet*(A) modules. IS26 insertions are important for plasmid evolution and the movement of resistance genes, particularly among *mcr*‐positive plasmids [41, 42]. The presence of IS26 and Tn3 in pEC0D1‐2 indicates its potential to acquire and rearrange resistance genes under antimicrobial selective pressure [43].

By contrast, plasmid pEC0D1‐3 carried both *bla*_*CTX-M−55*_ and *mcr-1.1*, each flanked by IS26 and ISApl1 elements, forming independent transposable modules. ISApl1 and IS26 elements were located adjacent to *mcr-1.1*, suggesting IS‐mediated recombination events. Unlike some previously reported *mcr-1*–carrying plasmids with different incompatibility backbones, pEC0D1‐3 belongs to the IncI2 incompatibility group, which is one of the most common plasmid types associated with *mcr-1* dissemination [44, 45]. This divergence implies that *mcr-1.1* has integrated into novel plasmid backbones, potentially broadening its host range. The presence of a complete transferable operon and T4SS supports its conjugative ability, consistent with the experimental confirmation of plasmid transfer to both *E. coli* and *K. pneumoniae* recipients. Comparative genomic analysis revealed >95% identity with clinical *K. pneumoniae* plasmids (e.g., pKP2016‐*CTX-M55-MCR1* and pKPN‐*MCR-55*), sharing an IncI2 backbone, transferable genes, and a complete T4SS. The results indicate that pEC0D1‐3 may have originated from or been exchanged with *K. pneumoniae* and may facilitate the transfer of resistance genes between *E. coli* and *K. pneumoniae*.

In summary, this study expands current knowledge of AMR in mink‐associated *E. coli* by identifying a transferable resistance gene combination of major clinical relevance. The coexistence of *bla*_CTX-M−55_ and *mcr-1.1*, the phylogenetic proximity to human clinical ST457 isolates, and the demonstrated plasmid transferability together indicate that mink may participate in broader AMR transmission networks. The emergence of transferable resistance determinants in mink‐associated *E. coli* indicates a potential route for their transmission between human and animal populations and highlights the need for continued surveillance.

## 5. Conclusion

A MDR *E. coli* strain EC0D1 was identified from farmed mink by whole genome sequencing which coharbored *bla*_CTX-M−55_ and *mcr-1.1* on conjugative plasmid pEC0D1‐3 and *tet*(A) and *floR* on plasmid pEC0D1‐2. Conjugation assays confirmed horizontal transfer of these resistance genes, mediating resistance to polymyxins, third‐generation cephalosporins, and tetracyclines. The results indicate that these plasmids have high transfer potential and can persist in different bacterial hosts suggesting that mink‐derived *E. coli* can act as a source of resistance genes. The emergence of a transferable plasmid coharboring *mcr-1.1* and *bla*_CTX-M−55_ in mink‐associated *E. coli* indicates an increasing risk of persistence and transmission across bacterial hosts. Further studies are required to clarify the transmission mechanisms and evolutionary characteristics of these resistance genes.

## Author Contributions

Shaohui Wang designed the study. Zizhu Lin performed the experiments, analyzed the data, and drafted the original manuscript. Zhiyang Wang assisted with the experiments. Xinyu Wang, Weiqi Guo, Jiangang Hu, Beibei Zhang, Haoheng Peng, Jingjing Qi, Mingxing Tian, Yanqing Bao, and Lei Deng contributed to data analysis, interpretation, and manuscript review. Shaohui Wang revised the manuscript and contributed to the experimental design. Haihua Li directed the study. Xue Bai provided strain resources.

## Funding

This work was supported by the National Natural Science Foundation of China (Grant 32302881), the Guangxi Key Research and Development Program (Grants FN2600640636 and AB23075145), the Shanghai Agricultural Science and Technology Innovation Project (Grant 2025‐02‐08‐00‐12‐F00042), the Central Public‐interest Scientific Institution Basal Research Fund (Grants Y2026YC46, 2026JB13, and 2026JB17), and the Agricultural Science and Technology Innovation Program (Grant CAAS‐ASTIP‐2026‐SHVRI).

## Disclosure

All authors have read and approved the final manuscript.

## Ethics Statement

All samples were derived from routine diagnostic submissions, and no animal procedures requiring ethical approval were conducted.

## Conflicts of Interest

The authors declare no conflicts of interest.

## Data Availability

The data of *E. coli* strain EC0D1 genome sequence are available in the GenBank: chr‐EC0D1 (https://www.ncbi.nlm.nih.gov/nuccore/CM129827.1), pEC0D1‐1 (https://www.ncbi.nlm.nih.gov/nuccore/NZ_JBRNXK010000002.1), pEC0D1‐2 (https://www.ncbi.nlm.nih.gov/nuccore/NZ_JBRNXK010000003.1), pEC0D1‐3 (https://www.ncbi.nlm.nih.gov/nuccore/NZ_JBRNXK010000004.1), pEC0D1‐4 (https://www.ncbi.nlm.nih.gov/nuccore/NZ_JBRNXK010000005.1), pEC0D1‐5 (https://www.ncbi.nlm.nih.gov/nuccore/NZ_JBRNXK010000006.1), pEC0D1‐6 (https://www.ncbi.nlm.nih.gov/nuccore/NZ_JBRNXK010000007.1), pEC0D1‐7 (https://www.ncbi.nlm.nih.gov/nuccore/NZ_JBRNXK010000008.1), and pEC0D1‐8 (https://www.ncbi.nlm.nih.gov/nuccore/NZ_JBRNXK010000009.1).

## References

[1] Ahmed S. K. , Hussein S. , and Qurbani K. , et al.Antimicrobial Resistance: Impacts, Challenges, and Future Prospects, Journal of Medicine, Surgery, and Public Health. (2024) 2, 10.1016/j.glmedi.2024.100081, 100081.

[2] Ramos S. , Silva V. , and Dapkevicius M. L. E. , et al. *Escherichia coli* as Commensal and Pathogenic Bacteria Among Food-Producing Animals: Health Implications of Extended Spectrum β-Lactamase (ESBL) Production, Animals. (2020) 10, no. 12, 10.3390/ani10122239, 2239. 33260303 PMC7761174

[3] Blum S. E. , Heller E. D. , Jacoby S. , Krifucks O. , and Leitner G. , Comparison of the Immune Responses Associated With Experimental Bovine Mastitis Caused by Different Strains of *Escherichia coli* , Journal of Dairy Research. (2017) 84, no. 2, 190–197, 10.1017/S0022029917000206. 28524018

[4] Tseng M. , Fratamico P. M. , Manning S. D. , and Funk J. A. , Shiga Toxin-Producing *Escherichia coli* in Swine: The Public Health Perspective, Animal Health Research Reviews. (2014) 15, no. 1, 63–75, 10.1017/S1466252313000170. 24397985 PMC4121380

[5] Kabir S. M. L. , Avian Colibacillosis and Salmonellosis: A Closer Look at Epidemiology, Pathogenesis, Diagnosis, Control and Public Health Concerns, International Journal of Environmental Research and Public Health. (2010) 7, no. 1, 89–114, 10.3390/ijerph7010089. 20195435 PMC2819778

[6] Zhao Y. , Guo L. , Li J. , Fang B. , and Huang X. , Molecular Epidemiology, Antimicrobial Susceptibility, and Pulsed-Field Gel Electrophoresis Genotyping of *Pseudomonas aeruginosa* Isolates From Mink, Canadian Journal of Veterinary Research–Revue Canadienne De Recherche Veterinaire. (2018) 82, no. 4, 256–263. 30363376 PMC6168023

[7] Qi J. , Li L. , and Du Y. , et al.The Identification, Typing, and Antimicrobial Susceptibility of *Pseudomonas aeruginosa* Isolated From Mink With Hemorrhagic Pneumonia, Veterinary Microbiology. (2014) 170, no. 3-4, 456–461, 10.1016/j.vetmic.2014.02.025. 24629901

[8] Telli A. E. , Telli N. , Biçer Y. , Turkal G. , Yılmaz T. , and Uçar G. C. , Co-Occurrence and Molecular Characterization of ESBL-Producing and Colistin-Resistant, *Escherichia coli*, Isolates From Retail Raw Meat, Foods. (2025) 14, no. 20, 10.3390/foods14203573, 3573. 41154109 PMC12564342

[9] Zhang Z. , Wei M. , Jia B. , and Yuan Y. , Recent Advances in Antimicrobial Resistance: Insights From *Escherichia coli* as a Model Organism, Microorganisms. (2025) 13, no. 1, 10.3390/microorganisms13010051, 51. PMC11767524 39858819

[10] Moran R. A. , Baomo L. , and Doughty E. L. , et al.Extended-Spectrum β-Lactamase Genes Traverse the, *Escherichia coli*, Populations of Intensive Care Unit Patients, Staff, and Environment, Microbiology Spectrum. (2023) 11, no. 2, 10.1128/spectrum.05074-22. PMC10100714 36916926

[11] Castanheira M. , Simner P. J. , and Bradford P. A. , Extended-Spectrum β-Lactamases: An Update on Their Characteristics, Epidemiology and Detection, JAC-Antimicrobial Resistance. (2021) 3, no. 3, 10.1093/jacamr/dlab092, dlab092. 34286272 PMC8284625

[12] Salman S. , Umar Z. , and Xiao Y. , Current Epidemiologic Features and Health Dynamics of ESBL-Producing *Escherichia coli* in China, Biosafety and Health. (2024) 6, no. 1, 40–49, 10.1016/j.bsheal.2024.01.002. 40078307 PMC11895021

[13] Wang C. , Song Y. , and Liang J. , et al.Antibiotic Resistance Genes are Transferred From Manure-Contaminated Water Bodies to the Gut Microbiota of Animals Through the Food Chain, Environmental Pollution. (2024) 363, 10.1016/j.envpol.2024.125087, 125087. 39383990

[14] Samtiya M. , Matthews K. R. , Dhewa T. , and Puniya A. K. , Antimicrobial Resistance in the Food Chain: Trends, Mechanisms, Pathways, and Possible Regulation Strategies, Foods. (2022) 11, no. 19, 10.3390/foods11192966, 2966. 36230040 PMC9614604

[15] Liu Y.-Y. , Wang Y. , and Walsh T. R. , et al.Emergence of Plasmid-Mediated Colistin Resistance Mechanism *MCR-1* in Animals and Human Beings in China: A Microbiological and Molecular Biological Study, The Lancet Infectious Diseases. (2016) 16, no. 2, 161–168, 10.1016/S1473-3099(15)00424-7. 26603172

[16] Wang C. , Feng Y. , Liu L. , Wei L. , Kang M. , and Zong Z. , Identification of Novel Mobile Colistin Resistance Gene *mcr-10* , Emerging Microbes & Infections. (2020) 9, no. 1, 508–516, 10.1080/22221751.2020.1732231. 32116151 PMC7067168

[17] Wang Z. , Zheng L. , and Zhu L. , et al.Co-Existence of *mcr-1* and *bla* _CTX−M_ From Porcine-Derived *Escherichia coli* Isolated in China and Selection of *mcr-1* Under Cephalosporins Pressure, Journal of Global Antimicrobial Resistance. (2025) 45, 164–172, 10.1016/j.jgar.2025.08.018. 40914242

[18] Hwang J. , Ye D.-Y. , Jung G. Y. , and Jang S. , Mobile Genetic Element-Based Gene Editing and Genome Engineering: Recent Advances and Applications, Biotechnology Advances. (2024) 72, 10.1016/j.biotechadv.2024.108343, 108343. 38521283

[19] Yu Y. , Hu B. , and Fan H. , et al.Molecular Epidemiology of Extraintestinal Pathogenic, *Escherichia coli*, Causing Hemorrhagic Pneumonia in Mink in Northern China, Frontiers in Cellular and Infection Microbiology. (2021) 11, 10.3389/fcimb.2021.781068, 781068. 34778114 PMC8581539

[20] Wang Z. , Wang X. , and Guo W. , et al.Identification and Genomic Analyses of a Multidrug Resistant Avian Pathogenic *Escherichia coli* Coharboring *mcr-1*, *bla* _(TEM−176)_ and *bla* _(CTX-M-‍14)_ Genes, Transboundary and Emerging Diseases. (2024) 2024, no. 1, 10.1155/2024/9332418, 9332418. 40303132 PMC12017198

[21] Chin C.-S. , Peluso P. , and Sedlazeck F. J. , et al.Phased Diploid Genome Assembly With Single-Molecule Real-Time Sequencing, Nature Methods. (2016) 13, no. 12, 1050–1054, 10.1038/nmeth.4035. 27749838 PMC5503144

[22] Walker B. J. , Abeel T. , and Shea T. , et al.Pilon: An Integrated Tool for Comprehensive Microbial Variant Detection and Genome Assembly Improvement, PLoS ONE. (2014) 9, no. 11, 10.1371/journal.pone.0112963. PMC4237348 25409509

[23] Alikhan N.-F. , Petty N. K. , Ben Zakour N. L. , and Beatson S. A. , BLAST Ring Image Generator (BRIG): Simple Prokaryote Genome Comparisons, BMC Genomics. (2011) 12, no. 1, 10.1186/1471-2164-12-402, 402. 21824423 PMC3163573

[24] Sullivan M. J. , Petty N. K. , and Beatson S. A. , Easyfig: A Genome Comparison Visualizer, Bioinformatics. (2011) 27, no. 7, 1009–1010, 10.1093/bioinformatics/btr039. 21278367 PMC3065679

[25] Cody A. J. , Bray J. E. , and Jolley K. A. , et al.Core Genome Multilocus Sequence Typing Scheme for Stable, Comparative Analyses of *Campylobacter jejuni* and, *C. coli*, Human Disease Isolates, Journal of Clinical Microbiology. (2017) 55, no. 7, 2086–2097, 10.1128/JCM.00080-17. 28446571 PMC5483910

[26] Schürch A. C. , Arredondo-Alonso S. , Willems R. J. L. , and Goering R. V. , Whole Genome Sequencing Options for Bacterial Strain Typing and Epidemiologic Analysis Based on Single Nucleotide Polymorphism Versus Gene-by-Gene-Based Approaches, Clinical Microbiology and Infection. (2018) 24, no. 4, 350–354, 10.1016/j.cmi.2017.12.016. 29309930

[27] Guenther S. , Ewers C. , and Wieler L. H. , Extended-Spectrum Beta-Lactamases Producing *E. coli* in Wildlife, Yet Another Form of Environmental Pollution?, Frontiers in Microbiology Review. (2011) 2, 246. 10.3389/fmicb.2011.00246 PMC3244693 22203818

[28] Vulfson L. , Pedersen K. , and Chriél M. , et al.Serogroups and Antimicrobial Susceptibility Among *Escherichia coli* Isolated From Farmed Mink (Mustela Vison Schreiber) in Denmark, Veterinary Microbiology. (2001) 79, no. 2, 143–153, 10.1016/S0378-1135(00)00343-6. 11230936

[29] Nikolaisen N. K. , Fertner M. , and Lassen D. C. K. , et al.Association Between Antibiotic Consumption and Resistance in Mink Production, Antibiotics. (2022) 11, no. 7, 10.3390/antibiotics11070927, 927. 35884181 PMC9311663

[30] Muloi D. M. , Hassell J. M. , and Wee B. A. , et al.Genomic Epidemiology of *Escherichia coli*: Antimicrobial Resistance Through a One Health Lens in Sympatric Humans, Livestock and Peri-Domestic Wildlife in Nairobi, Kenya, BMC Medicine. (2022) 20, no. 1, 10.1186/s12916-022-02677-7, 471. 36482440 PMC9730568

[31] Tacconelli E. , Carrara E. , and Savoldi A. , et al.Discovery, Research, and Development of New Antibiotics: The WHO Priority List of Antibiotic-Resistant Bacteria and Tuberculosis, The Lancet Infectious Diseases. (2018) 18, no. 3, 318–327, 10.1016/S1473-3099(17)30753-3. 29276051

[32] Shen Y. , Zhou H. , and Xu J. , et al.Anthropogenic and Environmental Factors Associated With High Incidence of *mcr-1* Carriage in Humans Across China, Nature Microbiology. (2018) 3, no. 9, 1054–1062, 10.1038/s41564-018-0205-8. PMC6198934 30038311

[33] Jarocki V. M. , Li D. , and Bogema D. R. , et al.Comparative Genomic Analysis of ESBL-Selected and Non-Selected *Escherichia coli* in Australian Wastewater: Elucidating Differences in Diversity, Antimicrobial Resistance, and Virulence Profiles, Science of The Total Environment. (2024) 949, 10.1016/j.scitotenv.2024.175079, 175079. 39094658

[34] Zhao X. , Chen H. , Bi W. , Shan H. , Wang J. , and Yang Z. , Coexistence and Genomics Characterization of *mcr-1* and Extended-Spectrum-β-Lactamase-Producing *Escherichia coli*, an Emerging Extensively Drug-Resistant Bacteria From Sheep in China, Science of The Total Environment. (2024) 955, 10.1016/j.scitotenv.2024.177016, 177016. 39426540

[35] Kim D. , Lee S. , and Hong J. S. , et al.Molecular Epidemiology of Extended-Spectrum β-Lactamase-Producing *Escherichia coli* in South Korea: A Korean Global Antimicrobial Resistance Surveillance System Report, Annals of Laboratory Medicine. (2026) 46, no. 1, 72–82, 10.3343/alm.2025.0145. 41189390 PMC12698250

[36] Silva A. , Silva V. , and Tavares T. , et al.Rabbits as a Reservoir of Multidrug-Resistant *Escherichia coli*: Clonal Lineages and Public Health Impact, Antibiotics. (2024) 13, no. 4, 10.3390/antibiotics13040376, 376. 38667052 PMC11047531

[37] Murray C. J. L. , Ikuta K. S. , and Sharara F. , et al.Global Burden of Bacterial Antimicrobial Resistance in 2019: A Systematic Analysis, The Lancet. (2022) 399, no. 10325, 629–655, 10.1016/S0140-6736(21)02724-0. PMC8841637 35065702

[38] Gorrie C. L. , Mirčeta M. , and Wick R. R. , et al.Genomic Dissection of *Klebsiella pneumoniae* Infections in Hospital Patients Reveals Insights Into an Opportunistic Pathogen, Nature Communications. (2022) 13, no. 1, 10.1038/s41467-022-30717-6, 3017. PMC9156735 35641522

[39] Komori K. , Aoki K. , and Harada S. , et al.Plasmid-Mediated Acquisition and Chromosomal Integration of *bla* _(CTX-M−14)_ in a Subclade of, *Escherichia coli*, ST131-H30 Clade C1, Antimicrobial Agents and Chemotherapy. (2024) 68, no. 9, 10.1128/aac.00817-24. PMC11373201 39133024

[40] Rozwandowicz M. , Brouwer M. S. M. , and Fischer J. , et al.Plasmids Carrying Antimicrobial Resistance Genes in Enterobacteriaceae, Journal of Antimicrobial Chemotherapy. (2018) 73, no. 5, 1121–1137, 10.1093/jac/dkx488. 29370371

[41] Harmer C. J. , Hall R. M. , and Detweiler C. S. , IS26 and the IS26 Family: Versatile Resistance Gene Movers and Genome Reorganizers, Microbiology and Molecular Biology Reviews. (2024) 88, no. 2, e00119–e00122, 10.1128/mmbr.00119-22. 38436262 PMC11332343

[42] Wu R. , Lv L. , and Wang C. , et al.IS26-Mediated Formation of a Hybrid Plasmid Carrying *mcr-‍1.1* , Infection and Drug Resistance. (2022) 15, 7227–7234, 10.2147/IDR.S390765. 36533252 PMC9748602

[43] Partridge S. R. , Kwong S. M. , Firth N. , and Jensen S. O. , Mobile Genetic Elements Associated With Antimicrobial Resistance, Clinical Microbiology Reviews. (2018) 31, no. 4, 10.1128/CMR.00088-17. PMC6148190 30068738

[44] Hu Z. , Chen D. , and Shi T. , et al.Antimicrobial Resistance of Enterobacteriaceae in Rabbit Farms: An Underestimated Reservoir Harboring *mcr-1.1* , Frontiers in Cellular and Infection Microbiology. (2025) 15, 10.3389/fcimb.2025.1663852, 1663852. 41104134 PMC12521101

[45] Gao X. J. , Yue C. , and Xiao L. D. , et al.Emergence of the IncHI2-ST3 Plasmid Co-Harbouring *bla* _(NDM−5)_, *mcr-1.1*, and *fosA3* in *Escherichia coli* From a Healthy Individual in China, International Journal of Antimicrobial Agents. (2025) 66, no. 6, 10.1016/j.ijantimicag.2025.107615, 107615. 40939666

